# Waste Glass-Derived Hierarchically Porous All-Inorganic Coatings for Sustainable Daytime Radiative Cooling

**DOI:** 10.3390/ma19071344

**Published:** 2026-03-28

**Authors:** Jiale Wang, Haiyang Chen, Weisu Weng, Wanfei Zhang, Boyu Qiao, Yu Xia, Yufan Liu, Ke Zhang, Mengyuan Du, Gaoxiang Ye, Jie Yan, Bin Li

**Affiliations:** 1 School of Civil and Engineering, Hebei University of Architecture, Zhangjiakou 075000, China15832311239@163.com (K.Z.); 13550894174@163.com (M.D.); yanjie6253@126.com (J.Y.); 2 Hebei Key Laboratory of Diagnosis, Reconstruction and Anti-Disaster of Civil Engineering, Zhangjiakou 075000, China; 3 Hebei Collaborative Innovation Center of Green Buildings, Zhangjiakou 075000, China; 4 Zhangjiakou Aocheng Investment Management Co., Ltd., Zhangjiakou 075000, China; haxzwf@163.com (W.Z.); 15075311190@163.com (B.Q.); 15076001124@163.com (Y.X.); 5 School of Materials Science and Engineering, University of Science and Technology Beijing, Beijing 100083, China

**Keywords:** passive daytime radiative cooling, waste glass upcycling, hierarchically porous network, all-inorganic coatings

## Abstract

**Highlights:**

**What are the main findings?**

- We developed an all-inorganic radiative cooling coating using upcycled construction waste glass and alumina via a low-temperature (600 °C) partial melting process.
- The optimized composite (50 wt.% glass/50 wt.% alumina) exhibits 96% solar reflectance, 95% infrared emittance, and peak net cooling powers of 108.1 W/m^2^.
- The material demonstrates extreme durability, retaining optical integrity after 1000 °C flame shocks, 60-day water immersion, and 80-day UV aging.

**What are the implications of the main findings?**

- Utilizing waste glass as a natural flux bypasses the high-cost, high-temperature sintering traditionally required for pure ceramic coolers.
- This study provides a highly scalable, environmentally sustainable, and commercially viable thermal management solution for energy-efficient buildings in harsh outdoor environments.

**Abstract:**

Passive daytime radiative cooling (PDRC) is a promising thermal management technology, yet its widespread application is hindered by the high production costs and poor durability of traditional organic-based materials. Here, we presented a hierarchically porous, all-inorganic PDRC coating synthesized from industrial waste glass and alumina microparticles via low-temperature (600 °C) processing. Rather than serving merely as a cheap substitute, the alkali oxides inherent in waste glass act as natural fluxes, enabling partial melting. Concurrently, the steric hindrance of alumina restricts full densification, spontaneously constructing a highly scattering random photonic network. The optimized composite (50 wt.% waste glass/50 wt.% alumina) achieves 96% solar reflectance and 95% atmospheric window emittance. Field tests confirmed sub-ambient cooling of ~4.0 °C (day) and ~4.5 °C (night), yielding a peak net cooling power of 108.1 W/m^2^. Accelerated weathering and thermal shock (1000 °C) tests demonstrated sustained optical stability under extreme environmental stress.

## 1. Introduction

Driven by rising global temperatures, rapid population growth, and accelerated urbanization, the utilization of air conditioning systems has surged, resulting in a dramatic increase in building energy consumption. According to the International Energy Agency (IEA), electricity consumption for space cooling accounts for approximately 20% of total global building energy usage, which corresponds to 10% of the world’s total electricity consumption [1,2]. Currently, nearly 5 billion people reside in regions with a strong demand for space cooling; however, fewer than one-third of these households possess air conditioning units. Projections indicate that the global energy demand for cooling will triple by 2050. Traditional cooling technologies, such as active air conditioning, are not only energy-intensive but also rely on refrigerants with high global warming potential (GWP), thereby exacerbating the climate crisis. Consequently, the development of zero-energy, environmentally friendly cooling technologies is imperative [3,4,5,6].

PDRC has emerged as a promising technology for achieving sub-ambient cooling, attributed to its high solar reflectance and capability to dissipate heat through the atmospheric transparency window [7,8]. This technology exhibits extensive application potential across various fields, including building energy efficiency [9,10,11], personal thermal management [12,13], and the thermal regulation of photovoltaic modules [14]. Given these broad applications, research in this domain has intensified, leading to the development of numerous polymer-based coatings, such as polyvinylidene fluoride (PVDF) and polydimethylsiloxane (PDMS) [15,16,17]. However, the inherent susceptibility of organic polymers to weathering and degradation limits their widespread adoption, particularly in long-term outdoor building applications. To address this limitation, researchers such as Toutam et al. [18] and Zhao et al. [19] have incorporated ceramic fillers, including SiO_2_ and TiO_2_, into polymer matrices. While these organic-inorganic composite coatings have improved weather resistance to a certain degree, the underlying durability issues associated with the organic matrix remain fundamentally unresolved. Therefore, the development of radiative cooling materials that possess exceptional weather resistance remains a significant scientific challenge.

Inorganic ceramic materials, such as SiO_2_, have garnered significant attention due to their intrinsic mechanical robustness and durability. Zhao et al. [20] demonstrated the substantial potential of ceramic materials (alumina and silica) for building radiative cooling. By structurally engineering these materials based on Mie-scattering theory, they achieved high solar reflectance and infrared emittance, thereby realizing effective daytime radiative cooling. Similarly, Lin et al. [21] presented a ceramic composite featuring a hierarchical porous structure, which attained a solar reflectance of 99.6% at a thickness of 600 μm. However, pure ceramic materials often suffer from poor mechanical integrity due to the lack of suitable binders, rendering them unsuitable for practical construction applications.

To address this challenge, Hu et al. [22] developed a random photonic composite consisting of a microporous glass framework embedded with Al_2_O_3_ particles. This coating achieved a sub-ambient cooling effect of 3.5 °C to 4 °C even under high-temperature and high-humidity conditions (80% relative humidity), demonstrating superior weatherability. Building upon this foundation, Xiao et al. [23] further optimized the particle size and loading content of the glass and alumina components (40–60 wt.% Al_2_O_3_) to ensure both structural stability and high solar reflectance (>0.95). Collectively, these studies validate the significant advantages of utilizing glass as a matrix combined with inorganic ceramic scatterers for radiative cooling applications.

Inorganic ceramic materials, such as pure SiO_2_ and Al_2_O_3_, possess intrinsic mechanical robustness and UV stability. By structurally engineering these materials based on Mie-scattering theory, previous studies have achieved substantial radiative cooling performance [20,21]. However, fabricating pure ceramic coolers typically requires demanding high-temperature sintering and costly processing to maintain the necessary hierarchical porosity.

**Figure 1:**
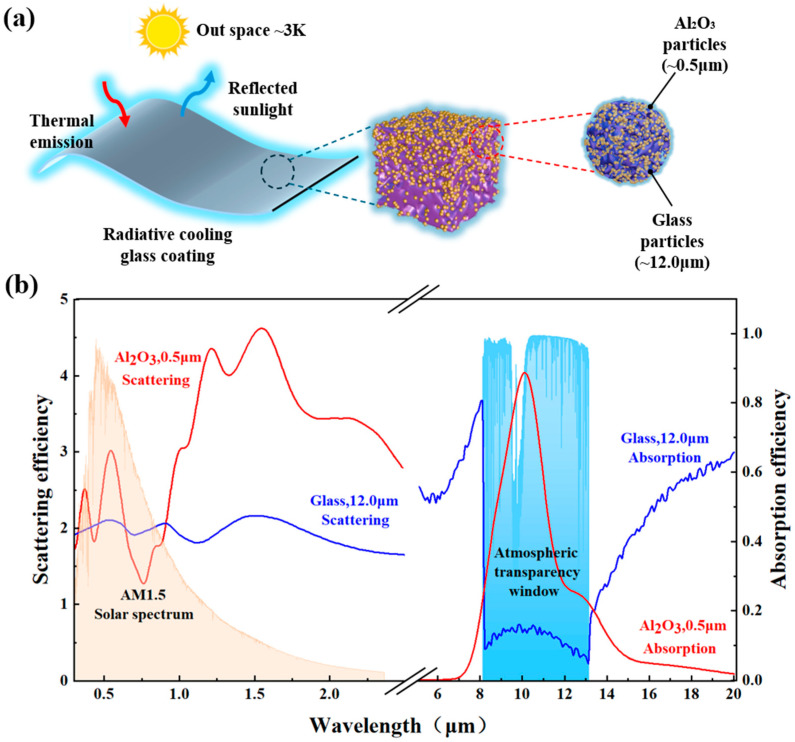
Composite cooling coatings: (**a**) schematic of radiative cooling mechanisms; (**b**) optical functionalities of glass and Al_2_O_3_ particles within the composite matrix. The scattering and absorption efficiencies of waste glass (blue line) and Al_2_O_3_ (red line) particles as functions of wavelength were calculated based on Lorenz–Mie theory. The dual-particle design optimizes material and dimensional parameters to enhance passive radiative cooling performance, particularly by achieving high reflectance within the solar spectrum and high emissivity within atmospheric transparency windows.

In this work, we present a hierarchically porous radiative cooling coating synthesized from a waste glass–alumina composite system (Figure 1a). Rather than treating waste glass merely as a low-cost substitute, we exploit its distinct chemical composition. The alkali and alkaline earth metal oxides (Na_2_O, CaO, etc.) inherent in construction waste glass serve as natural fluxes. This compositional feature lowers the softening point of the glass matrix, enabling low-temperature partial melting. During thermal treatment, the steric hindrance provided by the dispersed alumina microparticles restricts the flow of the molten glass, preventing full densification. This kinetic interplay naturally constructs a highly interconnected random photonic framework (Figure 1b). The resulting coating effectively manages broad-spectrum photon transport without requiring complex nanofabrication, achieving robust daytime radiative cooling alongside extreme environmental stability.

Crucially, by utilizing upcycled construction waste glass, this approach bypasses the high manufacturing costs typically associated with pure ceramic coolers. It provides a highly scalable, weather-resistant, and economically viable thermal management solution directly applicable to large-scale building envelopes, thereby demonstrating significant potential for commercial deployment in green building energy conservation.

## 2. Experimental Procedure

### 2.1. Materials

Industrial-grade α-Al_2_O_3_ powder was purchased from Wuhu Xinda New Materials Co., Ltd. (Wuhu, China). The waste glass utilized in this study was sourced from construction glass waste (Figure S1), with its corresponding TG-DSC curves presented in Figure S1b. Ethanol (analytical grade, 95%) was procured from Shanghai Macklin Biochemical Co., Ltd. (Shanghai, China). All materials were used as received without further purification.

### 2.2. Experimental Design

**Figure 2:**
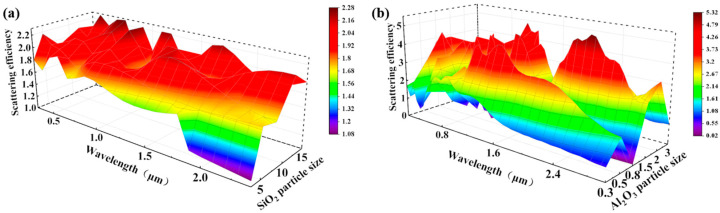
The scattering efficiency of different particle sizes under sunlight: (**a**) waste glass powder; (**b**) Al_2_O_3_ powder.

The primary raw material employed in this study was construction waste glass, the chemical composition of which is detailed in Table 1. Construction waste glass is a complex multi-phase system predominantly composed of a silica network. To establish a baseline for particle size selection, finite-difference time-domain (FDTD) numerical simulations were initially conducted using a simplified pure SiO_2_ model in an air medium. While the complex refractive index of waste glass differs slightly from pure silica due to metal oxide impurities, this simplified model provides critical qualitative guidance for optimizing scattering efficiency. Guided by these simulations and classical Lorenz–Mie theory, an average particle size of ~10 μm was targeted for the glass powder, alongside ~0.5 μm for the alumina component, to maximize impedance mismatch and backscattering in the solar spectrum. The simulation results concerning particle size are presented in Figure 2a and Figure S2.

Guided by Lorenz–Mie theory [24,25], the optimal particle size for the waste glass powder used in this experiment was determined to be approximately 10 μm. Similarly, Figure 2b and Figure S3 illustrate the simulation results for Al_2_O_3_ particles of different diameters. Based on these calculations, which align with the findings reported by Hu et al. [22], an average particle size of 0.5 μm was selected for the alumina component.

### 2.3. Sample Preparation

**Figure 3:**
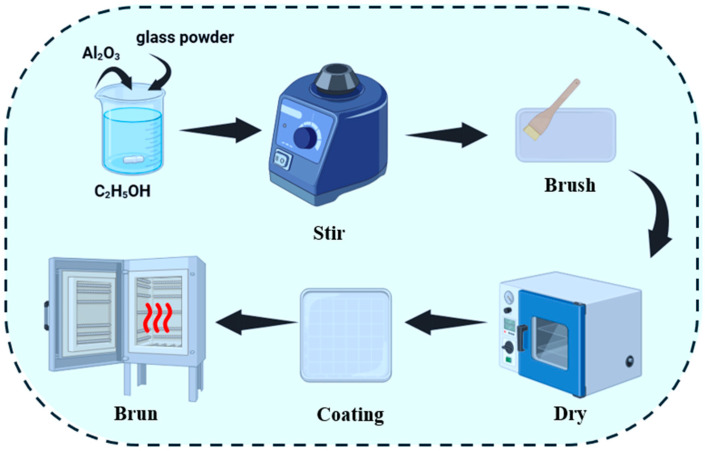
Schematic illustration of coating preparation.

The radiative cooling coating was prepared by mixing alumina and waste glass powder at various mass ratios. To this mixture, an appropriate amount of ethanol (C_2_H_5_OH) was added, followed by ball milling for 2 h (at a rotation speed of 300 rpm with a ball-to-powder mass ratio of 10:1) to ensure homogeneity. Subsequently, the resulting slurry was uniformly applied onto a ceramic tile substrate using a controlled volume-to-area brush application to achieve a target wet thickness and dried in an oven at 60 °C for approximately 3 min to strictly facilitate the complete evaporation of ethanol. Finally, the dried coating was subjected to heat treatment in a muffle furnace at 600 °C for less than 5 min (with a heating ramp rate of ~10 °C/min) to obtain the final radiative cooling coating (Figure 3).

### 2.4. Characterization

The microstructure of the coating was characterized using a scanning electron microscope (SEM, Apreo C, Thermo Fisher Scientific, Waltham, MA, USA) operating at an accelerating voltage of 10 kV, a working distance of approximately 10 mm, and a beam current of ~0.1 nA. Particle diameter distributions were subsequently analyzed from the SEM images using Nano Measure 1.2 software.

Optical properties were evaluated using two spectroscopic techniques. Spectral reflectance in the ultraviolet–visible region was measured with a UV-Vis spectrophotometer (Lambda 950, PerkinElmer, Shelton, CT, USA) over a wavelength range of 250–800 nm with a resolution of 1 nm. Infrared emittance was determined using a Fourier transform infrared (FTIR) spectrometer (Vertex 70v, Bruker, Mannheim, Germany) across a wavenumber range of 400–4000 cm^−1^ with a resolution of 4 cm^−1^.

Pore structure analysis, including pore size distribution and average pore diameter, was conducted using a high-performance automated mercury porosimeter (Auto Pore IV 9501, Micromeritics, Norcross, GA, USA). The analysis was performed under a pressure range of 0.5–33,000 psia, utilizing a mercury contact angle of 140° and a surface tension of 0.48 N/m.

Thermal stability was assessed by simultaneous Thermogravimetric Analysis (TGA) and Differential Scanning Calorimetry (DSC) using an SDT Q600 analyzer (TA Instruments, New Castle, DE, USA). Mass and thermal changes were recorded under a nitrogen atmosphere (flow rate: 50 mL/min) with a heating rate of 10 °C/min over a temperature range of 30–800 °C.

Ambient humidity and wind speed were monitored using a digital hygrometer (Testo 605i, Testo, Titisee-Neustadt, Germany) and a hot-wire anemometer (Kanomax 6004, KANOMAX, Osaka, Japan), respectively. Real-time temperature data were recorded using a multi-channel data logger (EX4000, Yili (Shenzhen) Technology Co., Ltd., Shenzhen, China) equipped with K-type thermocouples, featuring a sampling interval of 1 s and an accuracy of ±0.5 °C.

The testing apparatus consisted of thermocouples, a signal conversion module, and an aluminum foil-coated polystyrene foam box covered with a polyethylene (PE) film to house the test sample (5 cm × 5 cm). The entire assembly was elevated on a rack above a table to minimize thermal convection from the ground.

Accelerated weathering tests were conducted using a UV weathering test chamber (QUV/spray, Q-Lab, Westlake, OH, USA). Consistent experimental conditions were maintained throughout the process, specifically a temperature of 50 °C, a relative humidity of 50%, and an irradiance intensity of 0.68 W/m^2^ at 340 nm.

## 3. Results and Discussion

### 3.1. Composition and Microstructural Analysis

**Figure 4:**
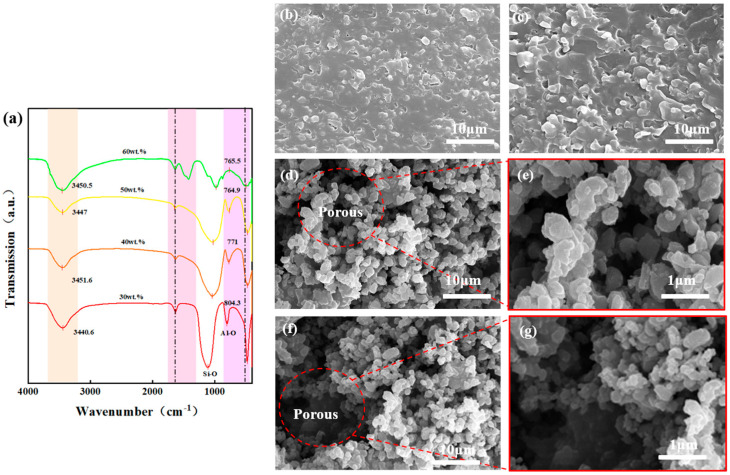
FTIR spectra and surface SEM micrographs of waste glass composite coatings with varying alumina mass fractions: (**a**) FTIR spectrum; (**b**) 30 wt.%; (**c**) 40 wt.%; (**d**,**e**) 50 wt. %; (**f**,**g**) 60 wt.%.

Figure 4a presents the FTIR spectra, which further confirms the chemical bond composition of the coating. The broad absorption band observed in the range of 1000–1100 cm^−1^ corresponds to the asymmetric stretching vibration of Si-O-Si bonds, while the absorption peak near 800 cm^−1^ was attributed to the vibration of Al-O bonds. These vibration modes aligned perfectly with the characteristic chemical features of waste glass and alumina.

Figure 4b–g display the SEM microstructures of the waste glass–alumina composite coatings with varying alumina concentrations. At an alumina loading of 30 wt.%, the surface appeared relatively dense, characterized by the presence of only a few distinct circular pores. The formation of this structure is primarily attributed to the high content of the glass phase; during the sintering process, the glass phase melts sufficiently to form a continuous matrix, effectively filling the interstitial voids between particles and leading to a densified overall structure. The isolated circular pores observed within the matrix originated mainly from gas bubbles released by the waste glass during the high-temperature sintering stage.

When the alumina content is increased to 40 wt.%, the micro-morphology shows no significant alteration. This indicates that within this composition range, the glass phase remains dominant; its melting behavior and fluidity continue to govern the densification trend of the sintering process. Consequently, the alumina particles act merely as a dispersed phase encapsulated within the continuous glass matrix, without yet exerting a decisive influence on the overall structural framework.

The microstructural evolution of the composites is highly dependent on the alumina mass fraction. At an optimal loading of 50 wt.% (Figure 4d,e), a pronounced hierarchically porous architecture formed. Mechanistically, at this specific ratio, the volume of the flux-softened glass phase is precisely insufficient to completely fill the interstitial voids between the alumina microparticles. Furthermore, the robust alumina skeleton exerts significant steric hindrance, arresting the viscous flow and coalescence of the molten glass. This restricted densification traps evolved during sintering and maintained open boundaries between the phases.

### 3.2. Optical Performance Analysis

**Figure 5:**
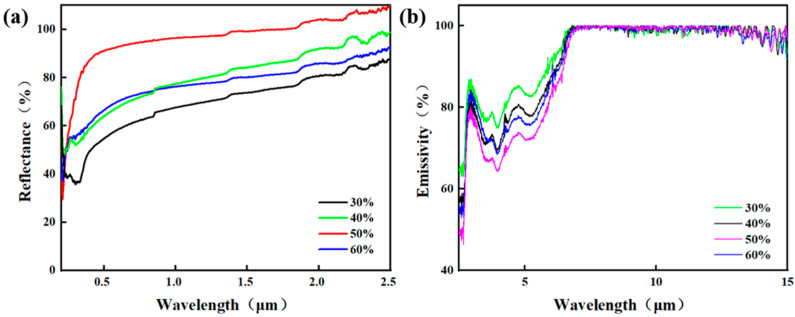
Optical properties of the coating: (**a**) Ultraviolet–visible spectroscopy; (**b**) Near-infrared spectroscopy.

To further investigate the influence of alumina content on the optical properties of the waste glass–alumina composite coatings, we conducted tests in the ultraviolet–visible (UV-Vis) and near-infrared (NIR) spectral regions, as shown in Figure 5. The UV-Vis analysis presented in Figure 5 reveals that the spectral curves for the 30 wt.% and 40 wt.% samples exhibit relatively low reflectance in the short-wavelength region (<1 μm), accompanied by certain fluctuations. This behavior is primarily attributed to the structural characteristics of these compositions: a continuous glass matrix with low porosity and a predominance of closed pores, which is consistent with the microstructural analysis in Figure 4. In this state, the contrast between the density of the scatterers and the refractive index is insufficient, resulting in limited scattering capability for short-wavelength light and allowing partial transmission or absorption. As the wavelength increases, scattering efficiency improves, leading to a rise in reflectance.

At a concentration of 50 wt.%, the reflectance reached its peak, and the overall curve was significantly elevated. This indicates that the alumina particles have formed a substantially continuous reflective layer, achieving an optimal balance of scattering and reflection efficiency for ultraviolet light. However, when the concentration is further increased to 60 wt.%, the curve initially rose but subsequently fell below that of the 40 wt.% sample. This reversal is likely due to an excess of solid particles leading to a denser sintered skeleton and a reduction in porosity from its peak level. Furthermore, the previously open, periodic pseudo-photonic crystal channel network may have partially collapsed, transitioning into more isolated, narrow, or irregular pores.

Figure 5b illustrates the near-infrared analysis of composites with varying alumina contents. At a concentration of 30 wt.%, the infrared emittance (absorptance) is relatively high. This stems from the lower alumina content, meaning the optical properties are dominated by the waste glass, which typically possesses strong thermal radiation (absorption) capabilities in the infrared band. When the concentration increases to 40 wt.%, the emittance begins to decline, suggesting that the addition of alumina particles initially enhances the coating’s ability to reflect infrared radiation.

At 50 wt.%, the emittance decreased significantly further, implying that the alumina particles have formed an effective infrared reflective barrier. The distribution of the porous structure effectively scatters and reflects thermal radiation, thereby suppressing the coating’s thermal emission (absorption) in this spectral band. However, at 60 wt.%, the downward trend in emittance slows or potentially reverses. This is often attributed to microstructural defects (such as micro-cracks or interfacial voids) caused by excessive particle content, which provide channels for infrared radiation penetration or emission, thereby reducing reflection efficiency.

In summary, the 50 wt.% waste glass–50 wt.% alumina coating exhibited the superior optical performance. This is evidenced by its maximal reflectance in the UV-NIR band, which effectively reflects solar radiation, combined with strong emittance in the far-infrared band that enhances self-radiative heat dissipation. Consequently, this composition achieves the optimal balance between spectral reflection and emission performance.

### 3.3. Mechanism Analysis

The exceptional optical properties of the coating are fundamentally rooted in its microstructural evolution. During the sintering process, the incorporation of a moderate concentration of alumina particles exerts a critical steric hindrance effect. This rigid skeleton not only suppresses the excessive flow and densification of the molten glass phase but also induces kinetic arrest. This phenomenon effectively prevents pore collapse, providing a structural foundation for the formation of a pseudo-photonic crystal architecture characterized by high poverty.

**Figure 6:**
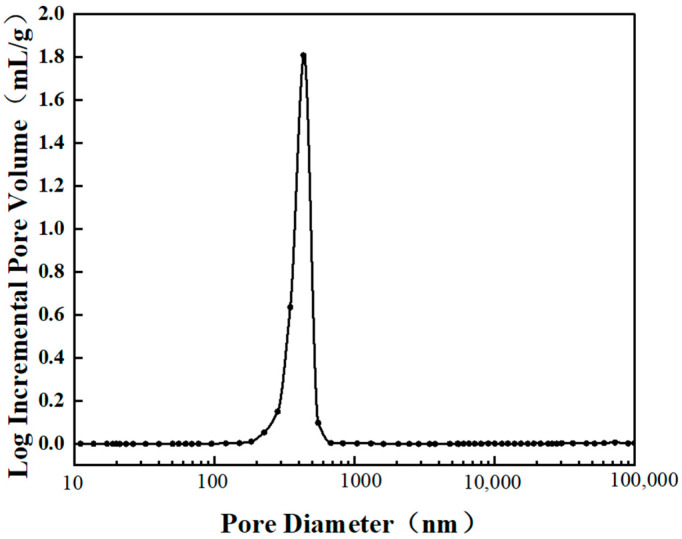
Pore size distribution diagram of a coating composed of 50 wt.% waste glass and 50 wt.% alumina.

The pore size distribution measured via mercury intrusion porosimetry (Figure 6) corroborates this mechanism, revealing a high concentration of micro- and nano-pores (300–600 nm) generated by kinetic arrest. These findings are in full agreement with the morphological evolution observed in SEM images (Figure 4). At the optimized 50 wt.% formulation, the material exhibits an optimal pore size distribution and superior connectivity, constructing an efficient porous framework for photonic management.

**Figure 7:**
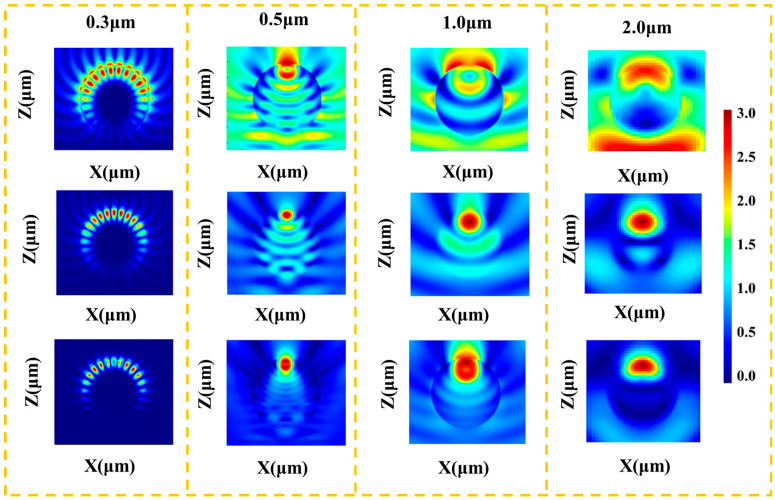
FDTD simulated normalized electric field distribution, temperature field, and power field along the boundary of the Z-X plane for porous structure models at different wavelengths.

To further elucidate the impact of porosity and validate the experimental results, FDTD simulations were conducted (Figure 7). The simulation reveals intense electric field enhancement (hotspots) at the pore edges and dielectric interfaces, driven by the sharp refractive index mismatch among air (*n* ≈ 1.0), glass (*n* ≈ 1.5), and alumina (*n* ≈ 1.7). This intense Mie scattering maximizes the scattering cross-sections, providing a robust physical justification for the peak solar reflectance observed in optical measurements.

Recent literature [26] highlights that in the ultra-low size-scale regime, classical Mie theory can deviate significantly from actual radiative properties due to non-local and quantum surface effects that differ from bulk materials. However, the scatterers in our hierarchically porous coating possess characteristic dimensions strictly in the micrometer range (approximately 0.5 μm for alumina and 10 μm for the glass matrix). At this scale, the materials operate well outside the deep-nanoscale regime. Therefore, the bulk complex refractive indices remain highly valid, and the classical Maxwell-Fresnel framework provides a robust and accurate physical description of the broadband scattering behaviors observed in our FDTD models.

Furthermore, the underlying physical mechanism of our daytime radiative cooler relies predominantly on pure dielectric scattering to reject solar heat, coupled with selective thermal emission. The pores and dielectric interfaces (glass/alumina/air) create severe impedance mismatches, functioning as highly efficient scattering centers (dielectric ‘hot spots’ of localized electric field enhancement) to reflect incident solar photons [22,26]. This mechanism contrasts fundamentally with other potential admixtures, such as plasmonic metallic nanoparticles. While plasmonic inclusions can generate intense, highly localized electromagnetic hot spots that are exceptionally efficient for photon absorption and subsequent re-emission, they intrinsically introduce strong parasitic absorption in the solar spectrum. For daytime sub-ambient cooling, minimizing solar heat gain is paramount. By utilizing wide-bandgap dielectrics, our coating circumvents the solar absorption penalties associated with plasmonic photon conversion, while simultaneously behaving as a strong broadband thermal emitter across the atmospheric transparency window.

In summary, by precisely engineering the composition and sintering conditions, this hierarchically porous structure achieves synergistic broadband solar reflectance and atmospheric window emittance, offering an ideal solution for daytime radiative cooling.

### 3.4. Outdoor Performance Analysis

As detailed in the Supplementary Information (Figure S4), the coating thickness significantly influences the solar reflectance of the 50 wt.% waste glass–50 wt.% alumina composites. To ensure statistical reliability, all optical and outdoor measurements were performed across multiple independent samples (*n* = 5). It is observed that at a thickness of ~150 μm, the solar reflectance was approximately 0.78 due to incomplete photon scattering. However, when the thickness increased to ~550 μm, the solar reflectance rose to 0.961. These results indicated that a coating thickness exceeding 500 μm was required to achieve high sub-ambient radiative cooling performance (reflectance > 0.95) under a solar irradiance of ~900 W/m^2^. Consequently, the coating thickness was controlled at approximately 550 μm for the subsequent experiments.

**Figure 8:**
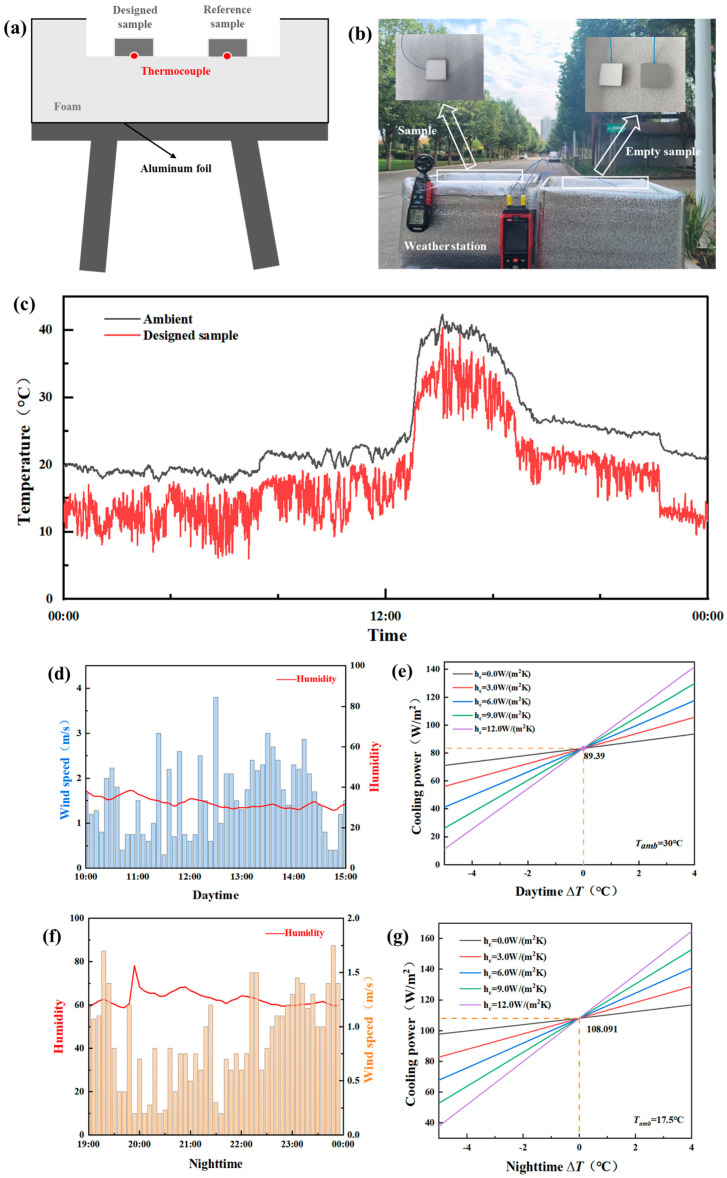
Outdoor coating testing: (**a**) schematic diagram of the setup for real-time measurement of cooling performance; (**b**) photograph of the setup for real-time measurement of cooling performance; (**c**) continuous monitoring of atmospheric and coating temperature variations throughout the day; (**d**) Variations in humidity and wind speed between 10:00 and 15:00 during daytime; (**e**) Net cooling power during daytime; (**f**) Variations in humidity and wind speed between 19:00 and 00:00 during nighttime; (**g**) Net cooling power during nighttime.

Figure 8a,b depict the experimental apparatus designed for the outdoor radiative cooling tests. The setup consists of a polystyrene foam box wrapped in aluminum foil to reflect sunlight and covered with a PE film. During testing, the apparatus was elevated on a table to minimize thermal interference from the ground and stabilize non-radiative heat exchange. As shown, a control experiment using a blank sample was conducted alongside the test sample to eliminate discrepancies caused by variations in airflow velocity.

Field tests were conducted at the Hebei Institute of Architecture and Civil Engineering in Zhangjiakou, Hebei Province, China (40.760156° N, 114.896884° E, altitude 716 m). We monitored the diurnal temperature variations in the coating applied to a ~1.5 mm thick ceramic tile, alongside the ambient temperature (Figure 8c). During midday (between 12:00 and 13:00), under a relative humidity of 30% and a wind speed of ~1.5 m/s, the temperature of the glass coating dropped approximately 4 °C below the ambient temperature of 30 °C. With a solar irradiance of ~900 W/m^2^, the net cooling power reached 89.39 W/m^2^ (Figure 8d). At night (between 19:00 and 21:00, Figure 8e), despite high humidity reaching 80% and a wind speed of 1.2 m/s, the glass coating achieved a temperature ~4.5 °C lower than the ambient temperature of 17.5 °C (Figure 8f), with a peak net cooling power of 108.1 W/m^2^ (Figure 8g). The observation that the temperature difference between the coating and the environment is greater at night than during the day can be rationally explained by the energy balance principle of radiative cooling (Equation (S6)). During the day, the net cooling power must overcome three major heat sources: direct solar radiation absorption (even with 96% reflectance, ~4% heat is absorbed), downward long-wave radiation from the warm atmosphere, and stronger convective heat transfer due to higher wind speeds. These factors collectively constrain the net heat dissipation capacity of the coating.

Conversely, at night, the most critical change is the elimination of solar heat input. Additionally, the lower atmospheric temperature weakens the downward atmospheric radiation, and lower wind speeds reduce heat gain from convection. Under these conditions, the coating can radiate heat to the cold outer space through the atmospheric window (8–13 μm) with minimal obstruction. These outdoor experiments confirmed that the developed glass coating exhibited superior radiative cooling performance during both day and night, even under high-humidity conditions.

This signifies that the coating possesses exceptional reflectivity within the solar spectrum while exhibiting a robust radiative heat dissipation capability in the mid-infrared (thermal radiation) band. This dual functionality allows for more effective suppression of temperature rise under direct sunlight.

**Figure 9:**
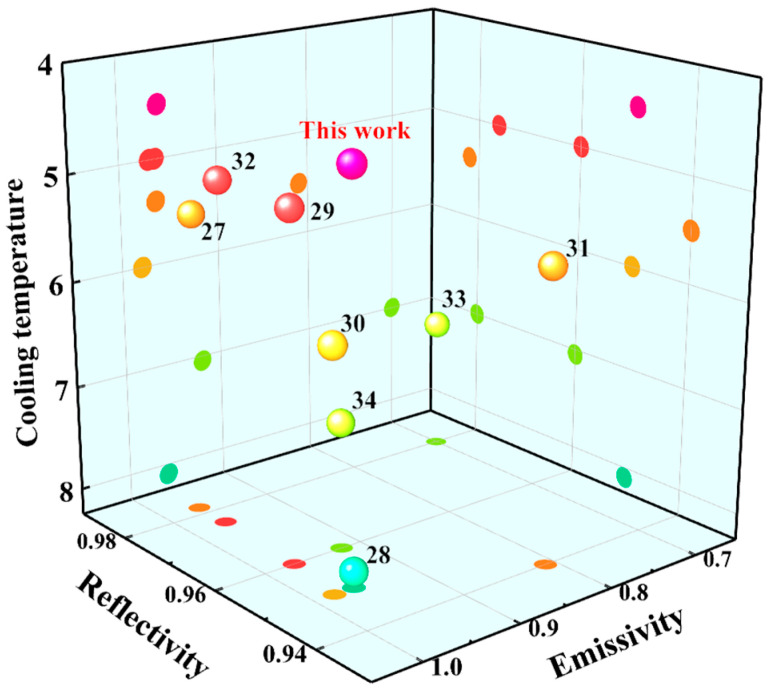
A comparative analysis of this study in relation to existing literature.

As illustrated in Figure 9, a 3D comparative analysis with other state-of-the-art radiative cooling materials is presented to highlight the specific novelty and contributions of this study. To fully contextualize these results, existing cooling materials must be evaluated on a spectrum of optical performance versus practical durability. Current literature predominantly features polymer-matrix coatings [27,28,29,30,31,32,33,34], which achieve high solar reflectance and infrared emittance; however, their organic nature makes them inherently susceptible to severe UV degradation and weathering over time. Conversely, while pure inorganic ceramic coolers offer excellent intrinsic mechanical robustness and UV stability, fabricating them typically requires highly energy-intensive sintering processes (>1000 °C) and costly fabrication to maintain the necessary hierarchical porosity.

What fundamentally sets our work apart is the resolution of this cost–durability paradox. As shown by the ‘This work’ data point in Figure 9, our optimized coating achieves a solar reflectance of 0.96 and an atmospheric window emittance of 0.95, placing its cooling performance strictly on par with advanced metamaterials and high-end polymers. More importantly, this step forward is achieved through a unique methodological contribution: rather than treating waste glass merely as a cheap, inert filler, we exploit the inherent alkali oxides as natural fluxes. This enables partial melting and the spontaneous formation of a highly scattering random photonic network at a significantly lowered processing temperature of just 600 °C. Consequently, our material provides the extreme environmental stability of pure ceramics while completely avoiding the high manufacturing costs and complex nanofabrication seen in prior studies, offering a highly scalable and superior alternative for real-world architectural applications.

### 3.5. Durability Analysis

**Figure 10:**
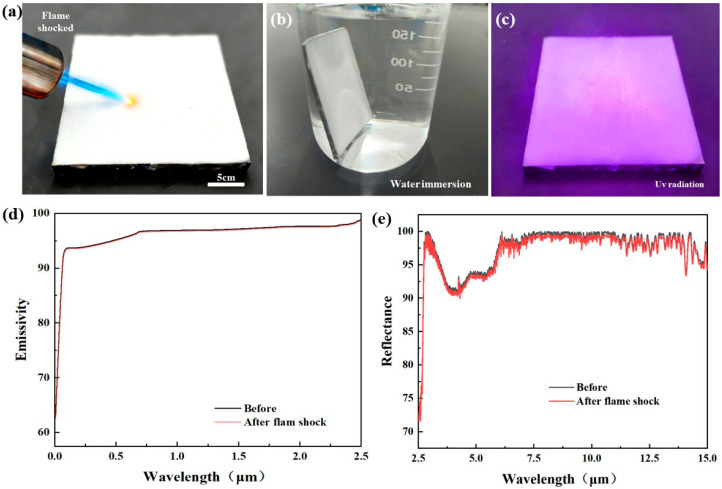
Environmental durability testing of coatings: (**a**) Photographs of flame impingement test; (**b**) Photographs of water immersion test; (**c**) Photographs of ultraviolet aging test; (**d**) Solar absorptance before and after high-temperature impact; (**e**) Solar reflectance before and after high-temperature impact.

To assess the long-term viability of the coating for practical applications, we evaluated its environmental stability under rigorous conditions. The coating exhibited excellent fire resistance, capable of withstanding a high-temperature flame shock of up to 1000 °C for approximately 10 s (Figure 10a). Crucially, the optical properties of the material showed no significant degradation following this intense thermal exposure (Figure 10e).

Typically, while the thermal emittance of radiative cooling materials remains relatively stable, their solar reflectance is highly sensitive to environmental factors, often degrading over time and consequently compromising passive cooling efficiency [35]. In contrast, our all-inorganic coating demonstrates remarkable durability.

As shown in Figure 10b,c, the coating was subjected to long-term weathering tests. Even after 60 days of water immersion followed by 80 days of accelerated UV irradiation, we observed virtually no decline in performance (Figure S5). Both the solar reflectance and thermal emittance exhibited negligible variations throughout these extended tests, confirming the superior weather resistance of the inorganic waste glass–alumina system.

## 4. Conclusions

In summary, we have successfully developed a robust, all-inorganic daytime radiative cooling coating utilizing upcycled industrial waste glass and alumina microparticles. Our main findings demonstrated that the optimized 50 wt.% waste glass/50 wt.% alumina composite achieved an outstanding solar reflectance of 0.96 and an atmospheric window emittance of 0.95. In practical field tests, this translated to sub-ambient temperature drops of ~4.0 °C during the day and ~4.5 °C at night, yielding a maximum net cooling power of 108.1 W/m^2^. Furthermore, unlike organic-based alternatives, our all-inorganic composite exhibited exceptional structural and optical stability when subjected to extreme environmental stresses, including 1000 °C flame thermal shocks, 60 days of water immersion, and 80 days of intense UV exposure. Ultimately, this work introduced a cost-effective, highly durable, and scalable materials strategy that transformed construction waste into a high-performance thermal management solution for sustainable buildings.

## Figures and Tables

**Table 1:** The composition and proportional content of various construction waste glass categories.

Materials	Na_2_O	MgO	Al_2_O_3_	SiO_2_	SO_3_	K_2_O	CaO	TiO_2_	Cr_2_O_3_	MnO
Sample A	16.266	1.420	3.025	68.594	0.090	0.668	8.312	0.075	0.072	0.044
Sample B	0.314	0.070	2.602	68.742	0.448	0.454	0.455	0.933	0.035	0.022
Sample C	15.922	4.005	1.162	69.935	0.270	0.262	7.808	0.005	0.027	0.025

## Data Availability

The original contributions presented in this study are included in the article/Supplementary Material. Further inquiries can be directed to the corresponding authors.

## Supplementary Materials

The following supporting information can be downloaded at: https://www.mdpi.com/article/10.3390/ma19071344/s1, Figure S1: Raw materials characterization: (a) Photograph of construction waste glass and its pulverized powder form; (b) TG-DSC curves of the waste glass powder. Figure S2: Normalized electric field distribution and power field on the Z-X plane boundary for silica particles of different diameters at various wavelengths. Figure S3: Normalized electric field distribution, temperature field, and power field on the Z-X plane boundary for alumina particles of different diameters at various wavelengths. Figure S4: Effect of radiative cooling glass coating thickness on solar reflectance, with an Al_2_O_3_ particle mass fraction of ~50 wt.%. Figure S5: Comparison of solar reflectance of the radiative cooling glass coating after (a) water immersion for 60 days; and (b) UV irradiation for 80 days. No significant change in solar reflectance was observed.

## References

[1] Zhang Q., Rao Z., Ma R. (2024). Radiative cooling: Arising from practice and in turn serving practice. Nanophotonics.

[2] Liang J., Wu J., Guo J., Li H., Zhou X., Liang S., Qiu C.W., Tao G. (2023). Radiative cooling for passive thermal management towards sustainable carbon neutrality. Natl. Sci. Rev..

[3] Zhao D., Aili A., Zhai Y., Xu S., Tan G., Yin X., Yang R. (2019). Radiative sky cooling: Fundamental principles, materials, and applications. Appl. Phys. Rev..

[4] Liu J., Tang H., Jiang C., Wu S., Ye L., Zhao D., Zhou Z. (2022). Micro-nano porous structure for efficient daytime radiative sky cooling. Adv. Funct. Mater..

[5] Zeyghami M., Goswami D.Y., Stefanakos E. (2018). A review of clear sky radiative cooling developments and applications in renewable power systems and passive building cooling. Sol. Energy Mater. Sol. Cells.

[6] Zhao B., Hu M., Ao X., Chen N., Pei G. (2019). Radiative cooling: A review of fundamentals, materials, applications, and prospects. Appl. Energy.

[7] Chan Y.H., Zhang Y., Tennakoon T., Fu S.C., Chan K.C., Tso C.Y., Yu K.M., Wan M.P., Huang B.L., Yao S. (2022). Potential passive cooling methods based on radiation controls in buildings. Energy Convers. Manag..

[8] Ziaeemehr B., Jandaghian Z., Ge H., Lacasse M., Moore T. (2023). Increasing Solar Reflectivity of Building Envelope Materials to Mitigate Urban Heat Islands: State-of-the-Art Review. Buildings.

[9] Feng S., Yao L., Feng M., Cai H., He X., He M., Bu X., Zhou Y., Zhang T. (2024). Sustainable regeneration of waste polystyrene foam as cooling coating: Building cooling energy saving, CO_2_ emission reduction and costbenefit prospective. J. Clean. Prod..

[10] Zhu F.L., Feng Q.Q. (2021). Recent advances in textile materials for personal radiative thermal management in indoor and outdoor environments. Int. J. Therm. Sci..

[11] Jiang Q., Wan Y., Li X., Qu X., Ouyang S., Qin Y., Zhu Z., Wang Y., He H., Yu Z. (2024). Fabrication of thermal insulation sodium alginate/SiO_2_ composite aerogel with superior radiative cooling function for firefighting clothing. Pigm. Resin Technol..

[12] Zheng Y., Fu Z., Sun X., Zhou L., Wei X., Zhang J., Xia W., Liu Y. (2024). Al_2_O_3_/graphene/PVDF-HFP radiative cooling coating reinforced heat dissipation of BIPV modules. J. Appl. Polym. Sci..

[13] Chen C., Zhao B., Wang R., He Z., Wang J.L., Hu M., Li X.L., Pei G., Liu J.W., Yu S.H. (2022). Janus Helical Ribbon Structure of Ordered Nanowire Films for Flexible Solar Thermoelectric Devices. Adv. Mater..

[14] Cui Y., Luo X., Zhang F., Sun L., Jin N., Yang W. (2022). Progress of passive daytime radiative cooling technologies towards commercial applications. Particuology.

[15] Liu P., Sun Y., Huang X., Guo Z. (2023). Colorful Superhydrophobic Composite Coating for Efficient Passive Radiation Cooling. Ind. Eng. Chem. Res..

[16] Liu H., Wang F., Lei S., Ou J., Li W. (2021). Large-area fabrication of colorful superhydrophobic coatings with high solar reflectivity. Constr. Build. Mater..

[17] Du T., Niu J., Wang L., Bai J., Wang S., Li S., Fan Y. (2022). Daytime Radiative Cooling Coating Based on the Y_2_O_3_/TiO_2_ Microparticle-Embedded PDMS Polymer on Energy-Saving Buildings. ACS Appl. Mater. Interfaces.

[18] Toutam V., Jain P., Sharma R., Bathula S., Dhar A. (2015). Radius ratio rule for surface hydrophilization of polydimethyl siloxane and silica nanoparticle composite. Appl. Surf. Sci..

[19] Zhao Y., Liu Y., Xu Q., Barahman M., Lyons A.M. (2015). Catalytic, self-cleaning surface with stable superhydrophobic properties: Printed polydimethylsiloxane (PDMS) arrays embedded with TiO_2_ nanoparticles. ACS Appl. Mater. Interfaces.

[20] Zhao D., Tang H. (2023). Staying stably cool in the sunlight. Science.

[21] Lin K., Chen S., Zeng Y., Ho T.C., Zhu Y., Wang X., Wang X., Huang B., Chao C.Y.H., Wang Z. (2023). Hierarchically structured passive radiative cooling ceramic with high solar reflectivity. Science.

[22] Zhao X., Li T., Xie H., Liu H., Wang L., Qu Y., Srebric J., Hu L. (2023). A solution-processed radiative cooling glass. Science.

[23] Xiao R., Dai X., Zhong J., Ma Y., Jiang X., He J., Wang Y., Huang B. (2024). Toward waste glass upcycling: Preparation and characterization of high-volume waste glass geopolymer composites. Sustain. Mater. Technol..

[24] Mätzler C. (2002). MATLAB Functions for Mie Scattering and Absorption.

[25] Zhao X., Mofid S.A., Jelle B.P., Tan G., Yin X., Yang R. (2020). Optically switchable thermally insulating VO_2_-aerogel hybrid film for window retrofits. Appl. Energy.

[26] Jacak J.E., Jacak W.A. (2025). Plasmons in metallic nanoclusters exhibit nonharmonic phenomena. Phys. Rev. A.

[27] Park H., Joo B.S., Kang G., Ko H., Kim J.H. (2025). A hierarchically engineered polymer composite with a dual-scatter structure for enhanced passive radiative cooling. J. Mater. Chem. A.

[28] Song X., Gao Y., Zhang P. (2024). Optical properties of the polymeric radiative cooler with embedded nano/micro-particles. Renew. Sust. Energy Rev..

[29] Jin C., Zhang W., Ni J., Li L., Hao Y., Pei G., Zhao B. (2025). Multi-interface porous coating for efficient sub-ambient daytime radiative cooling. Sol. Energy Mat. Sol. Cells.

[30] Wang T., Wu Y., Shi L., Hu X., Chen M., Wu L. (2021). A structural polymer for highly efficient all-day passive radiative cooling. Nat. Commun..

[31] Yu H., Lu J., Yan J., Bai T., Niu Z., Ye B., Cheng W., Wang D., Huan S., Han G. (2025). Selective Emission Fabric for Indoor and Outdoor Passive Radiative Cooling in Personal Thermal Management. Nano-Micro Lett..

[32] Xiong L., Chen C., Tian K., Zhang X., Wen M., Guo C., Cheng M., Li Q., Fu Q., Deng H. (2025). Dielectric Aggregation-Mediated Dual-Band Robust Optical Performance for Low-Cost Radiative Cooling. Adv. Mater..

[33] Wang L., Zheng Z., Gou Y., Liang W., Yu W. (2021). Fabry–Perot resonance assisted dual-layer coating with enhanced wavelength-selective refection and emission for daytime radiative cooling. Opt. Commun..

[34] Jeon S.K., Kim J.T., Kim M.S., Kim I.S., Park S.J., Jeong H., Lee G.J., Kim Y.J. (2023). Scalable, Patternable Glass-Infiltrated Ceramic Radiative Coolers for Energy-Saving Architectural Applications. Adv. Sci..

[35] Takebayashi H., Miki K., Sakai K., Murata Y., Matsumoto T., Wada S., Aoyama T. (2016). Experimental examination of solar reflectance of high-reflectance paint in Japan with natural and accelerated aging. Energy Build..

