# Supplementary material for: Waste Glass-Derived Hierarchically Porous All-Inorganic Coatings for Sustainable Daytime Radiative Cooling
Source: Materials (Basel). 2026 Mar 28;19(7):1344. doi: 10.3390/ma19071344 (PMC13073652; doi:10.3390/ma19071344)
Supplement: Supplementary file 1 [file materials-19-01344-s001.zip › materials-4213882-supplementary.pdf]

Supplementary Information

Supplementary methods

1. Theoretical calculation of scattering and absorption properties for glass and Al_2O_3 particles

In 1908, Gustav Mie derived the rigorous analytical solution for the elastic scattering of electromagnetic waves by a homogeneous dielectric sphere by solving Maxwell's equations. This formalism, known as Lorenz-Mie scattering theory, enables the calculation of scattering characteristics for spherical particles of arbitrary size and material. Based on this theory, the optical efficiencies are expressed as:

$$Q_{sca} = \frac{2}{x^2}\sum_{n=1}^{\infty}(2n+1)(|a_n|^2+|b_n|^2) \tag{S1}$$

$$Q_{ext} = \frac{2}{x^2}\sum_{n=1}^{\infty}(2n+1)\mathrm{Re}(a_n+b_n) \tag{S2}$$

$$Q_{abs} = Q_{ext} - Q_{sca} \tag{S3}$$

Where Q_{ext}、Q_{sca} and Q_{abs} denote the extinction, scattering, and absorption efficiency factors of the particles, respectively. The size parameter is defined as x = kD/2, where D is the particle diameter, and k=2π/λ is the wavenumber, with λ being the wavelength of the incident radiation. Re[·] denotes the real part of the complex number. The terms a_n and b_n are the Mie coefficients, which are functions of the particle size, the wavelength of incident radiation, and the complex refractive index of the simulated particles.

2. Average solar reflectance and infrared emissivity within the transparent atmospheric window

The spectral average solar reflectance γ is evaluated using the spectral solar irradiance of Air Mass 1.5 Global （$I_{sol,\lambda}$，AM1.5） as the weighting factor, as given by the following equation:

$$\gamma = \frac{\int_{0.3\mu m}^{2.5\mu m}\gamma_\lambda I_{sol,\lambda}d\lambda}{\int_{0.3\mu m}^{2.5\mu m}I_{sol,\lambda}d\lambda} \tag{S4}$$

Where γ_λ represents the spectral reflectance in the solar region. Similarly, the spectral average absorption efficiency α within the 8-13μm range can be evaluated using the spectral blackbody emissive power as the weighting factor. This is given by:

$$\alpha = \frac{\int_{8\mu m}^{13\mu m} \alpha_\lambda I_{b\lambda} d\lambda}{\int_{8\mu m}^{13\mu m} I_{b\lambda} d\lambda} \tag{S5}$$

Where $I_{b\lambda} = \frac{2hc_0^2}{\lambda^5[\exp(hc_0/\lambda k_b T)-1]}$, Here, h = 6.626 × 10^{-34} J·s is the Planck constant, k_b = 1.381 × 10^{-23}J/K is the Boltzmann constant, and c_0 = 2.998 × 10^8 m/s is the speed of light in a vacuum.

3. Theoretical modeling of radiative cooling performance

The net cooling power P_{net}（T）of a surface exposed to solar radiation and a clear sky can be expressed as:

$$P_{net}(T) = (1 - R_{solar})P_{sun} + P_{conv+cond}(T, T_{amb}) + P_{atm}(T_{amb}) - P_{rad}(T) \tag{S6}$$

Where T represents the surface temperature, T_{amb} is the ambient temperature (i.e., air temperature near the ground), and R_{solar} denotes the average solar reflectance of the surface:

$$R_{solar} = \frac{\int_0^{\frac{\pi}{2}} \int_{0.3\mu m}^{2.5\mu m} R(\lambda,\theta) \cos\theta I_{AM1.5}(\lambda) d\lambda d\theta}{\int_{0.3\mu m}^{2.5\mu m} I_{AM1.5}(\lambda) d\lambda} \tag{S7}$$

In this equation, $R(\lambda, \theta)$ = 1.0 − ε（λ，θ）represents the spectral solar reflectance （0.3–2.5μm）, where θ is the angle between the incident solar radiation and the surface normal (i.e., the zenith angle), and ε（λ， θ）represents the surface emissivity as a function of wavelength and directional angle. $I_{AM1.5}(\lambda)$ refers to the Air Mass 1.5 (AM 1.5) solar spectrum. P_{sun} is the power density of solar irradiance reaching the panel surface, calculated as:

$$P_{sun} = \int_0^\infty I_{solar}(\lambda) d\lambda \tag{S8}$$

Where I_{solar}（λ）represents the spectral solar radiation, including both direct and diffuse components. $P_{conv+cond}$ represents the heat gain received from the surroundings via conduction and convection, calculated as follows:

$$P_{conv+cond}(T, T_{amb}) = h(T_{amb} - T) \tag{S9}$$

Where h = 8.3 + 2.5v represents the comprehensive heat transfer coefficient between the panel surface and the surrounding environment in W/（m^2·K）, with v denoting the wind speed (m/s). P_{atm} represents the radiative heating received from atmospheric thermal radiation, expressed as:

$$P_{atm} = \int d\Omega \cos\theta \int_0^\infty \epsilon(\lambda,\theta)\epsilon_{atm}(\lambda,\theta,PW) I_b(\lambda, T_{amb}) d\lambda \tag{S10}$$

Where Ω represents the hemispherical solid angle, with $\int d\Omega = \int_0^{\pi/2} \mathrm{d}\theta \sin\theta$, ϵ_{atm} is the atmospheric emissivity, which is a function of the surface zenith angle, wavelength, and atmospheric Precipitable Water (PW). $I_{b\lambda}$ is the spectral intensity of blackbody radiation.

The cooling power is significantly influenced by factors such as atmospheric transmittance, humidity, wind speed, and solar irradiance (T_{amb}=303K).

4. Durability testing

Here, we utilize the indicators provided by the equipment manufacturer to calculate the conversion between the testing time in the UV accelerated weathering tester and outdoor illumination conditions. Recognizing that outdoor lighting conditions vary with season and region, the equivalent outdoor exposure time for one day of UV testing is estimated using the following equations:

$$\frac{Q_{year}}{365 \times 24} = Q_{hour} \tag{S11}$$

$$\frac{T_{set}}{T_{amb}} \times \frac{Q_a}{Q_{hour}} \times 1.5 = D_{outdoor} \tag{S12}$$

Where Q_{year} represents the total annual solar radiation; Q_{hour} represents the calculated annual average hourly solar irradiance; Q_a denotes the total radiation intensity set for the test; T_{set} is the temperature set for the test; T_{amb} refers to the local annual average temperature; and $D_{outdoor}$ represents the duration of outdoor exposure.

In our experiment, based on data for Hebei Province, Q_{year} is 1200 kW h^{-1} m^{-2} and T_{amb} is 12 °C. The test parameters were set as Q_a= 0.7 kW m^{-2} and T_{set}= 25 °C. Upon calculation, the $D_{outdoor}$ duration is determined to be 16 days. Therefore, the 72-hour UV irradiation test conducted in this experiment corresponds to 48 days of outdoor exposure.

Supplementary figures：

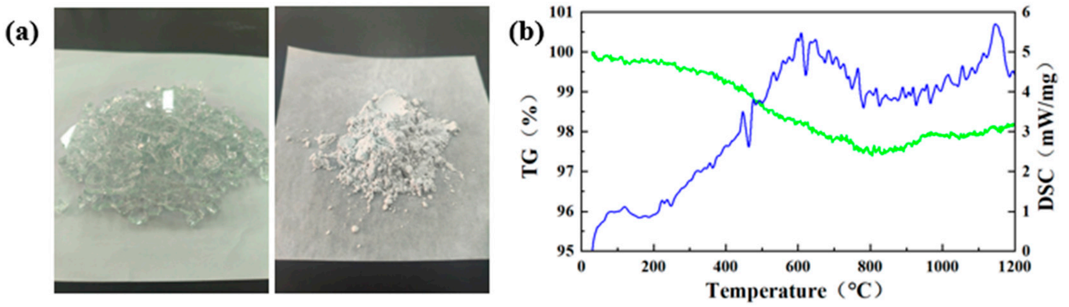

Fig.S1 Raw materials characterization: (a) Photograph of construction waste glass and its pulverized powder form; (b) TG-DSC curves of the waste glass powder.

In the TG curve, a distinct mass loss step is observed in the range of 100-200°C, with a weight loss of approximately 3%, primarily attributed to the desorption of physically adsorbed water on the glass surface and the decomposition of organic residues. The DSC curve exhibits two distinct thermal events: the first is an endothermic peak at approximately 100°C, corresponding to the glass transition temperature of the glass; the second is an exothermic peak at approximately 500°C, corresponding to the softening and viscous flow process of the waste glass particles.

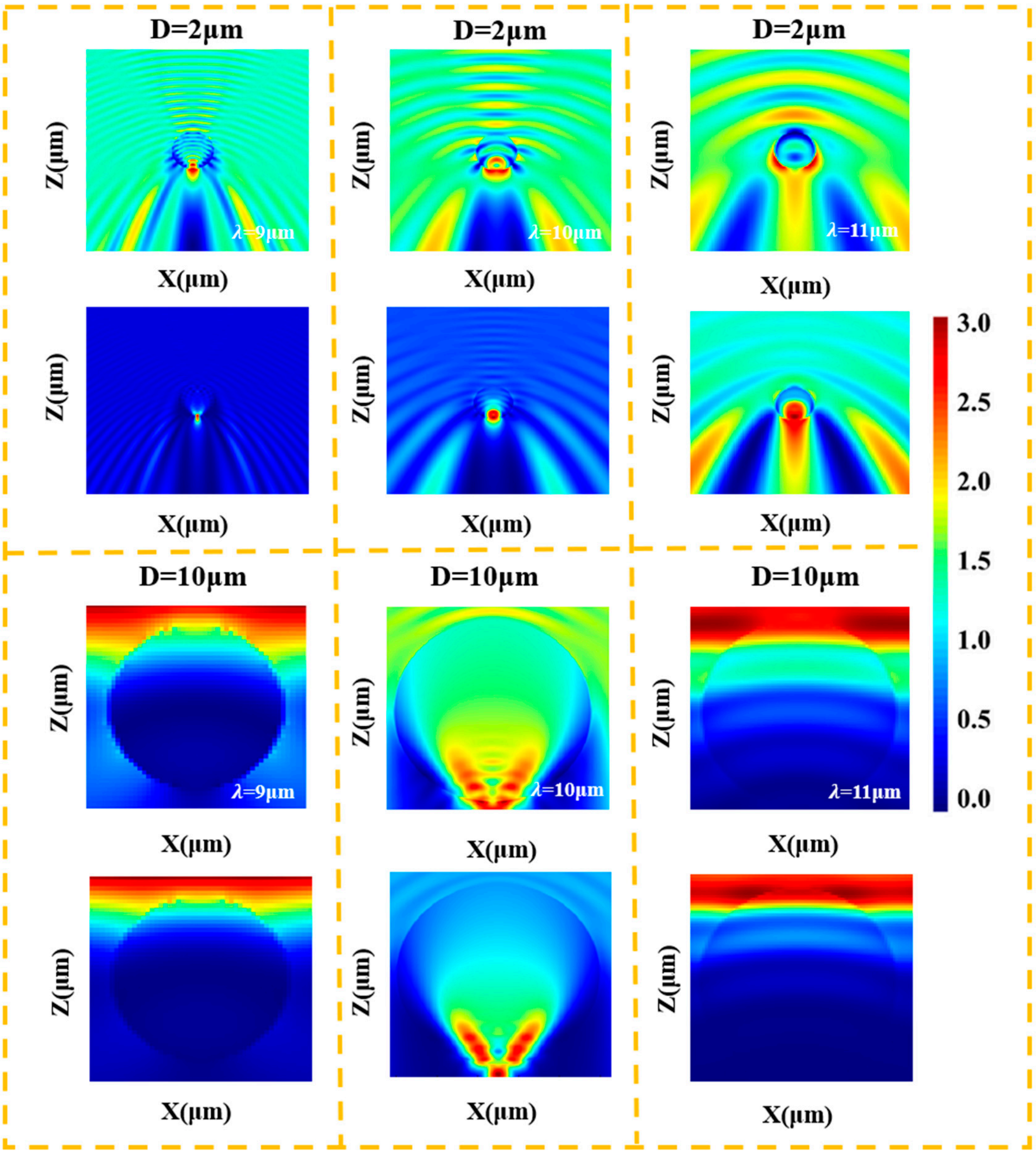

Fig.S2 Normalized electric field distribution and power field on the Z-X plane boundary for silica particles of different diameters at various wavelengths.

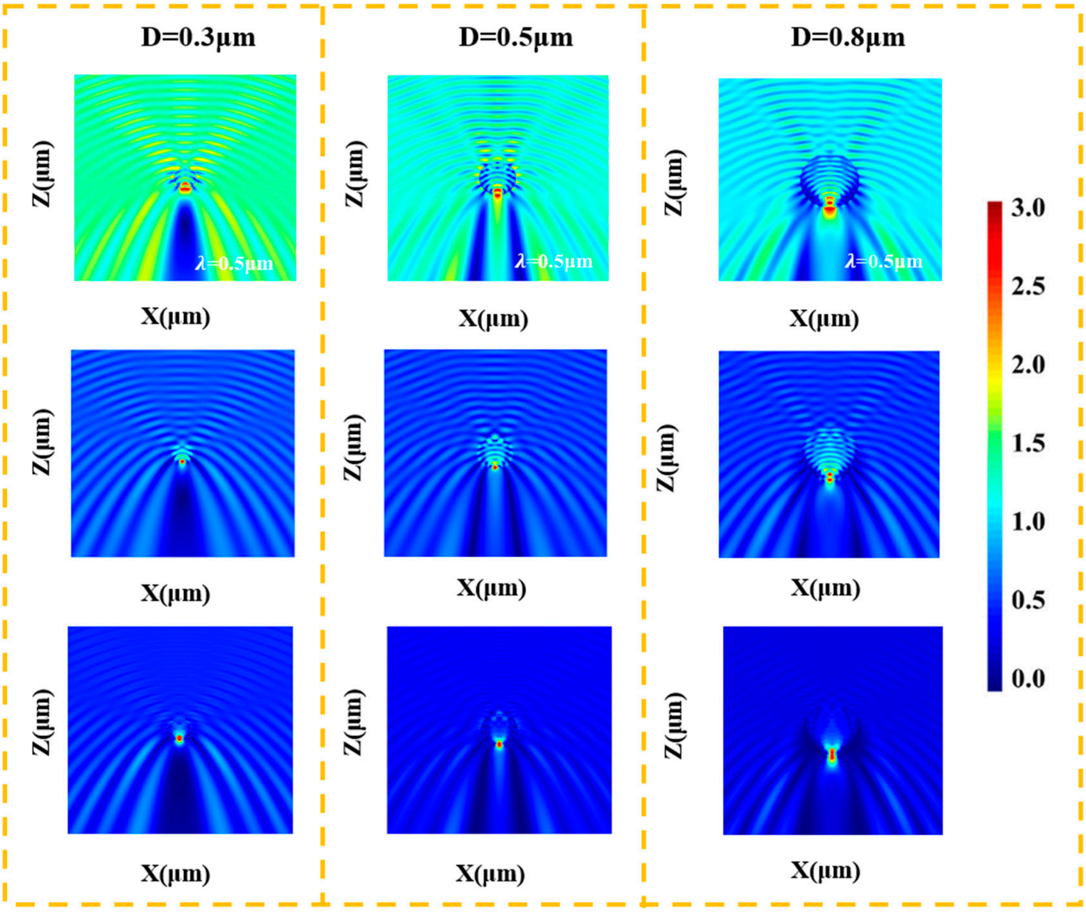

Fig.S3 Normalized electric field distribution, temperature field, and power field on the Z-X plane boundary for alumina particles of different diameters at various wavelengths.

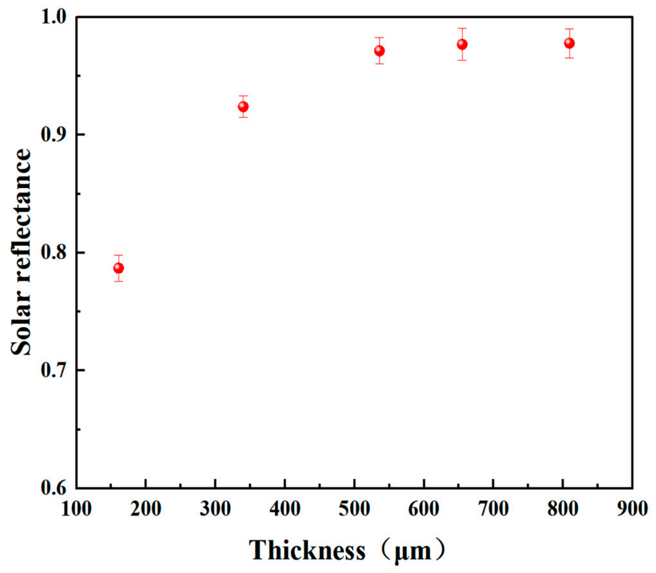

Fig.S4 Effect of radiative cooling glass coating thickness on solar reflectance, with an Al_2O_3 particle mass fraction of ~50 wt.%.

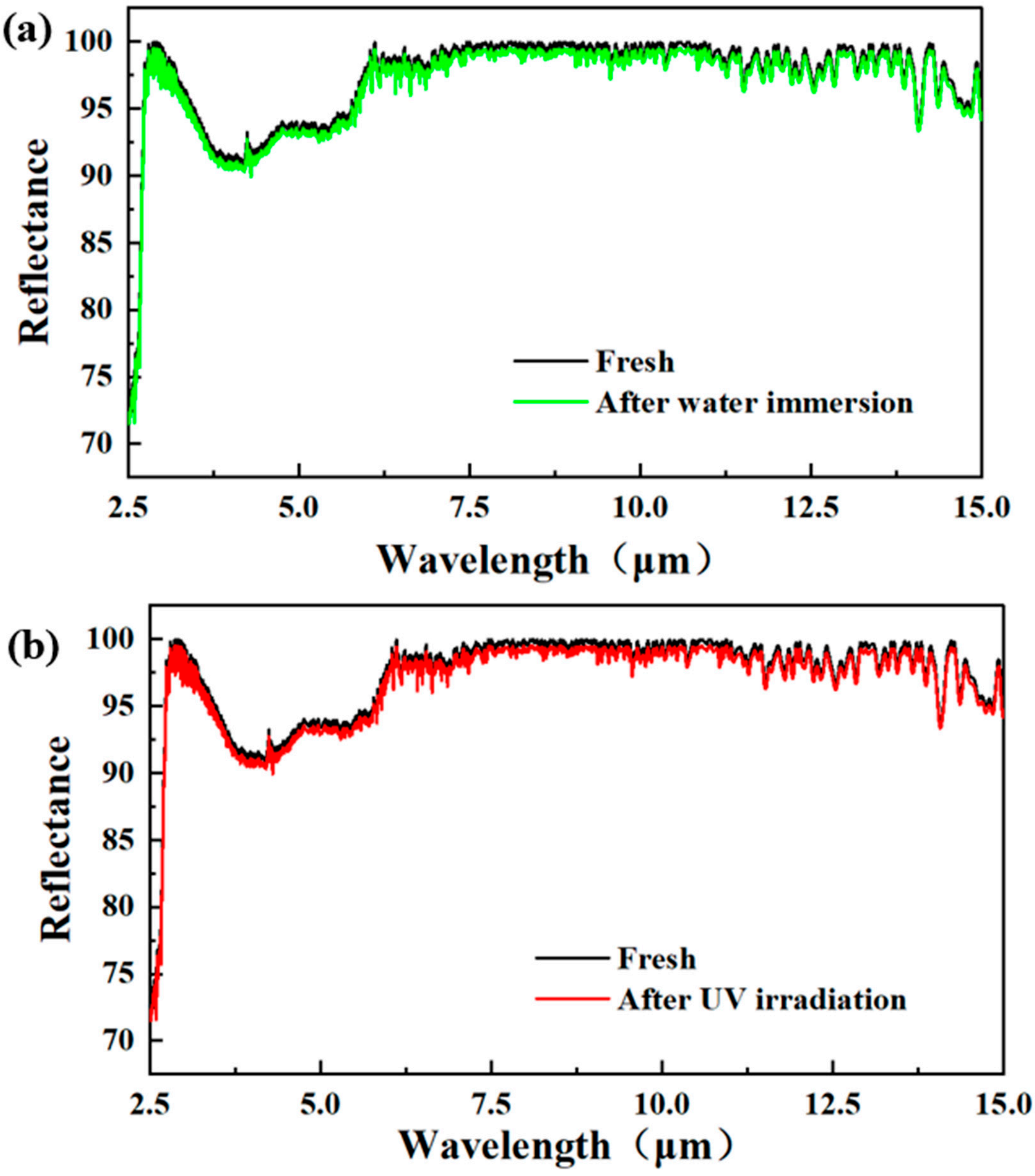

Fig.S5 Comparison of solar reflectance of the radiative cooling glass coating after (a) water immersion for 60 days; and (b) UV irradiation for 80 days. No significant change in solar reflectance was observed.
